# Asymmetric Dual-Interface Passivation with Functionalized Ammonium Halides for High-Performance Inverted CsPbI_2_Br Perovskite Solar Cells

**DOI:** 10.3390/nano16130795

**Published:** 2026-06-27

**Authors:** Xin Liu, Chengguo Liu, Wei Li, Wangyang Song, Xiaoxuan Li, Bo Li, Kun Zhao, Shu Wang, Jie Li, Dingyu Yang

**Affiliations:** Optoelectronic Sensor Devices and Systems Key Laboratory of Sichuan Provincial Universities, Sichuan Meteorological Optoelectronic Sensor Technology and Application Engineering Research Center, Information Materials and Device Applications Key Laboratory of Sichuan Provincial Universities, College of Optoelectronic Engineering (Chengdu IC Valley Industrial College), Chengdu University of Information Technology, Chengdu 610225, China; 16608310283@163.com (C.L.); liwei_5966@163.com (W.L.); 18328570344@163.com (W.S.); lixiaoxuan_5158@163.com (X.L.); 15700414911@163.com (B.L.); 15808447847@163.com (K.Z.); wangshudameinv@outlook.com (S.W.); lijie@cuit.edu.cn (J.L.)

**Keywords:** inverted perovskite solar cells, CsPbI_2_Br, interfacial passivation, ammonium halides, asymmetric passivation

## Abstract

Interfacial defect passivation has emerged as a critical strategy for mitigating non-radiative recombination losses in inorganic perovskite solar cells (PSCs). However, the distinct chemical environments at the bottom (hole-transport layer) and top (electron-transport layer) interfaces demand passivation agents with tailored functionalities—a principle that remains largely underexplored. Herein, we systematically employed two organic ammonium iodide salts, phenylethylammonium iodide (PEAI) and 2-thiophenemethylammonium iodide (ThMI), to separately modulate the bottom NiO_x_/CsPbI_2_Br and top CsPbI_2_Br/PCBM interfaces of inverted PSCs with a configuration of ITO/NiO_x_/CsPbI_2_Br/PCBM/BCP/Ag. We reveal different interfacial modulation effects: bottom-interface modification by both PEAI and ThMI dramatically improves the fill factor (FF), with PEAI delivering a more pronounced enhancement due to improved interfacial contact and reduced series resistance. However, top-interface passivation effectively boosts the open-circuit voltage (V_oc_), where ThMI exhibits superior voltage elevation capability over PEAI by neutralizing undercoordinated Pb^2+^ defects via its thiophene moiety. Capitalizing on this complementary selectivity, we construct an asymmetric dual-interface passivation architecture with PEAI at the bottom and ThMI at the top (ITO/NiO_x_/PEAI/CsPbI_2_Br/ThMI/PCBM/BCP/Ag), which synergistically enhances both FF and V_oc_. Consequently, the optimized PEAI/ThMI device achieves a champion power conversion efficiency (PCE) of 15.44%, with a V_oc_ of 1.15 V, a J_sc_ of 16.34 mA/cm^2^, and an FF of 82.15%, significantly outperforming the control device (11.79%). This work establishes a rational design paradigm for interface-specific passivation in inverted inorganic PSCs, highlighting the importance of molecular functionality in addressing distinct interfacial recombination pathways.

## 1. Introduction

Organic–inorganic hybrid perovskite solar cells (PSCs) have undergone unprecedented development over the past decade, with certified power conversion efficiencies (PCEs) now exceeding 26%, owing to their strong light absorption, high charge-carrier mobility, and solution processability [1,2,3]. Nevertheless, the intrinsic thermal instability of hybrid perovskites, stemming from the volatile nature of organic cations (MA^+^, FA^+^), remains a critical obstacle to their commercialization [4,5]. By contrast, all-inorganic cesium lead halide perovskites (CsPbX_3_) completely eliminate organic components and exhibit exceptional thermal stability, thus attracting intense research interest [4,6,7]. Among these, CsPbI_2_Br offers an optimal balance between light-harvesting capability and phase stability, featuring a bandgap of ~1.90 eV, which renders it highly attractive for both single-junction devices and as a top subcell in silicon-based tandem photovoltaics [8,9,10,11].

Despite these advantages, CsPbI_2_Br perovskite films are intrinsically prone to a high density of ionic defects, particularly iodine vacancies and undercoordinated Pb^2+^ species at grain boundaries and surfaces, as well as to unfavorable energy level alignment at charge-transport interfaces. These shortcomings provoke severe non-radiative charge recombination and substantial energy losses, resulting in unsatisfactory V_oc_ and FF [12,13]. Hence, improving V_oc_ and FF remains a major challenge for further advancing the performance of CsPbI_2_Br PSCs [14]. Interfacial defect passivation coupled with energy level engineering has proven to be one of the most effective strategies to tackle these issues [15,16]. Organic ammonium halide salts, such as phenylethylammonium iodide (PEAI), fluorinated salts, and alkylammonium iodides, are widely employed as interfacial passivators because of their strong coordination affinity toward perovskite surface defects [17,18,19]. Previous reports have demonstrated that PEAI can passivate undercoordinated Pb^2+^ defects through electrostatic interactions and simultaneously optimize interfacial contact, thereby markedly reducing non-radiative recombination [20,21]. In the realm of all-inorganic CsPbI_2_Br solar cells, Zhang et al. employed hexyltrimethylammonium bromide to reduce the energy offset at the perovskite/carbon interface, leading to a certified efficiency of 14.0% [22]. Meanwhile, Luo et al. proposed a hierarchical passivation strategy using EDEA at the bottom and PBAI at the top interfaces (glass/ITO/ZnO/reference or EDEA-based CsPbI_2_Br/Spiro-OMeTAD/MoO_3_/Ag), which reduced trap densities and boosted the PCE to 16.6% [23]. However, most studies have focused on single-interface modification, and the differential modulation effects on the bottom NiO_x_/perovskite interface versus the top perovskite/electron transport layer (PCBM) interface remain poorly understood.

The chemical and electronic environments of the buried bottom (HTL/perovskite) and top (perovskite/ETL) interfaces are fundamentally different. The bottom interface critically governs hole extraction and is susceptible to recombination due to energy misalignment and poor wettability, whereas the top interface dictates electron extraction. Consequently, a “one-size-fits-all” passivation strategy is likely suboptimal. Recent work has highlighted the promise of heterocyclic-containing molecules, such as thiophene derivatives, as passivators because the electron-rich sulfur atom can strongly coordinate with Pb^2+^ ions [24,25]. This suggests that introducing specific side groups into ammonium halides could impart interfacial selectivity. Compared to benzene-based PEAI, 2-thiophenemethylammonium iodide (ThMI) contains electron-rich sulfur atoms in the thiophene ring, which can form stronger Lewis acid–base coordination with unsaturated Pb^2+^ than benzene groups [26]. Nevertheless, the application of ThMI in all-inorganic CsPbI_2_Br PSCs and its interfacial modulation mechanism have rarely been explored. More importantly, the synergistic effect of PEAI and ThMI on dual-interface engineering, as well as their respective advantages in regulating V_oc_ and FF, has yet to be systematically investigated.

Herein, we propose an asymmetric dual-interface passivation strategy. We systematically study the passivation effects of PEAI and ThMI applied individually to either the bottom NiO_x_/perovskite interface, the top perovskite/PCBM interface, or both, in inverted PSCs with the architecture ITO/NiO_x_/CsPbI_2_Br/PCBM/BCP/Ag. Our results reveal a clear functional selectivity: bottom-interface modification with either PEAI or ThMI dramatically improves FF, with PEAI yielding a more pronounced enhancement, owing to improved interfacial contact and reduced series resistance. In contrast, top-interface passivation predominantly boosts V_oc_, and ThMI exhibits superior voltage-elevation capability over PEAI by effectively neutralizing undercoordinated Pb^2+^ defects through its thiophene moiety. Using this complementary selectivity, we construct a heterologous dual-modified device with PEAI at the bottom and ThMI at the top (ITO/NiO_x_/PEAI/CsPbI_2_Br/ThMI/PCBM/BCP/Ag), which synergistically enhances both FF and V_oc_. Consequently, the optimized PEAI/ThMI device achieves a champion PCE of 15.44% with a V_oc_ of 1.15 V, a J_sc_ of 16.34 mA/cm^2^, and an FF of 82.15%, substantially outperforming the control device (11.79%). Comprehensive morphological, spectroscopic, and electrical characterizations confirm the distinct passivation mechanisms and the synergistic effect of dual modification in reducing defect density, prolonging carrier lifetime, and improving environmental stability. This work establishes a rational design paradigm for interface-specific passivation in inverted inorganic PSCs, underscoring the importance of molecular functionality in addressing distinct interfacial recombination pathways.

## 2. Results and Discussion

**Figure 1:**
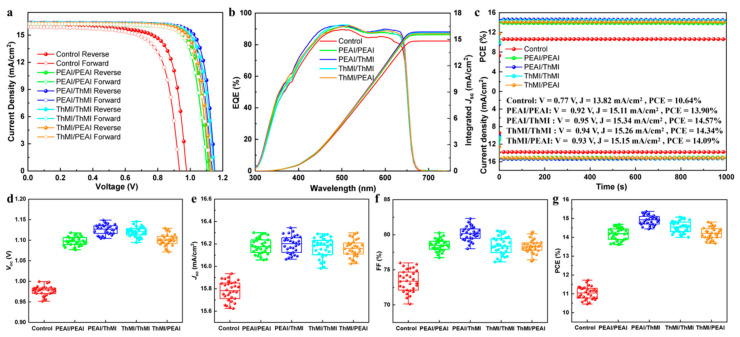
Photovoltaic performance of the control and dual-surface-passivated CsPbI_2_Br PSCs. (**a**) Forward and reverse J–V curves of the champion devices measured under simulated AM1.5G illumination (100 mW/cm^2^). (**b**) EQE spectra. (**c**) Steady-state photocurrent and PCE. Statistical distributions of the photovoltaic parameters: (**d**) V_oc_, (**e**) J_sc_, (**f**) FF, and (**g**) PCE.

To systematically evaluate the distinct roles of PEAI and ThMI, inverted PSCs with the architecture ITO/NiO_x_/Bottom-passivator/CsPbI_2_Br/Top-passivator/PCBM/BCP/Ag were fabricated. The passivators were introduced either at the bottom (NiO_x_/perovskite) interface, the top (perovskite/PCBM) interface, or both (detailed in Table 1). Figure 1a presents the current density-voltage (J–V) characteristics of the champion devices under simulated AM 1.5G illumination (100 mW/cm^2^), with corresponding parameters summarized in Table 1. The control device exhibited a PCE of 11.79%, with a V_oc_ of 0.98 V, a J_sc_ of 15.94 mA/cm^2^, and an FF of 75.49%. Bottom-interface modification with PEAI (ITO/NiO_x_/PEAI/CsPbI_2_Br) predominantly enhanced the FF to 81.20% (PCE 13.48%), while the V_oc_ showed a modest increase to 1.02 V (Table S4). This improvement is attributed to PEAI’s ability to passivate undercoordinated Pb^2+^ defects at the buried NiO_x_/perovskite interface, reducing hole extraction barriers and shunt pathways [27]. However, top-interface passivation with ThMI (ITO/NiO_x_/CsPbI_2_Br/ThMI) significantly boosted the V_oc_ to 1.15 V (PCE 14.76%, Table S3), with a comparatively lower FF improvement (78.61%). ThMI’s superior surface defect passivation effectively suppresses interfacial non-radiative recombination, directly enhancing the V_oc_ [13]. Notably, the dual-interface passivation strategy employing PEAI at the bottom and ThMI at the top (PEAI/ThMI) yielded the best-performing device, achieving a champion PCE of 15.44%, with a V_oc_ of 1.15 V, a J_sc_ of 16.34 mA/cm^2^, and an exceptional FF of 82.15%. The control device exhibits a pronounced hysteresis index (HI) of 9.84%, which is attributed to the high density of ionic defects, particularly iodine vacancies and undercoordinated Pb^2+^ species at the perovskite surfaces and grain boundaries. These defects facilitate ion migration and charge trapping/detrapping processes under electric field bias, leading to the well-known anomalous J–V hysteresis in perovskite solar cells. Upon asymmetric dual-interface passivation with PEAI at the bottom and ThMI at the top, the HI is dramatically reduced to 1.55%. This suppression arises from the synergistic defect passivation at both interfaces. PEAI improves the buried NiO_x_/perovskite contact and reduces interfacial trap states, while ThMI effectively neutralizes surface undercoordinated Pb^2+^ defects via its electron-rich thiophene moiety, thereby immobilizing halide ions and suppressing ion migration. The combined effect minimizes charge accumulation and mitigates the capacitive response, resulting in nearly hysteresis-free device operation [28].

The external quantum efficiency (EQE) spectra are shown in Figure 1b. The integrated J_sc_ from the EQE for the champion PEAI/ThMI device was 15.86 mA/cm^2^, which is within 5% of the value obtained from the J–V measurement, confirming the accuracy of the light illumination calibration [29]. Short-wavelength (300–500 nm) light is primarily absorbed near the bottom NiO_x_/CsPbI_2_Br interface. Bottom-interface passivation with either PEAI or ThMI improves the perovskite film quality (larger grains, fewer pinholes, reduced interfacial defects) and enhances interfacial contact, thereby lowering hole extraction barriers and suppressing recombination at the buried interface. Consequently, devices with bottom passivation exhibit higher short-wavelength EQE. The slight difference in EQE intensity between PEAI and ThMI is attributed to their distinct influences on film morphology and interfacial energy level alignment. Long-wavelength (500–700 nm) light penetrates deeper into the perovskite film and is absorbed closer to the top CsPbI_2_Br/PCBM interface. ThMI contains an electron-rich sulfur atom in its thiophene ring, which can strongly coordinate with undercoordinated Pb^2+^ defects on the perovskite surface, effectively neutralizing these trap states [25]. This strong passivation effect greatly suppresses interfacial non-radiative recombination and facilitates electron extraction into PCBM. Therefore, devices with ThMI top passivation show markedly enhanced long-wavelength EQE compared to PEAI-modified or control devices. This interpretation is consistent with the stronger photoluminescence quenching and longer carrier lifetimes observed for ThMI-based top passivation. Furthermore, the steady-state power output measured at the maximum power point (Figure 1c) stabilized at 14.57%, demonstrating reliable steady-state power output of the champion device under continuous illumination for 1000 s [30]. Statistical distributions of the photovoltaic parameters (V_oc_, J_sc_, FF, and PCE) obtained from 30 individual devices for each condition are presented in Figure 1d–g. The PEAI/ThMI dual-passivation strategy not only delivered the highest average PCE but also exhibited the narrowest distribution and smallest standard deviation, indicating superior reproducibility compared to single-interface or control devices. This statistical analysis solidifies the conclusion that the synergistic combination of bottom and top passivation layers is a robust and effective approach for fabricating high-performance inverted CsPbI_2_Br PSCs.

**Figure 2:**
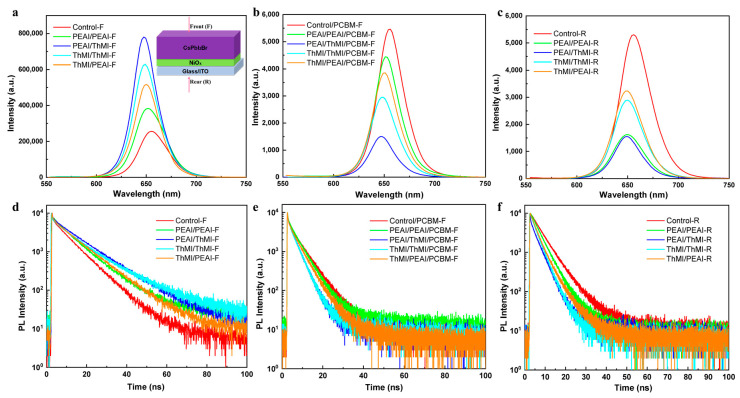
Steady-state PL spectra of (**a**) control, PEAI/PEAI, PEAI/ThMI, ThMI/ThMI, and ThMI/PEAI films under front-side excitation; (**b**) control/PCBM, PEAI/PEAI/PCBM, PEAI/ThMI/PCBM, ThMI/ThMI/PCBM, and ThMI/PEAI/PCBM films under front-side excitation; and (**c**) the same films as in (**a**) under rear-side excitation. Insets show the corresponding excitation geometries. TRPL decay curves recorded under identical excitation configurations: (**d**) front-side (without PCBM), (**e**) front-side (with PCBM), and (**f**) rear-side.

To elucidate the origin of the functional selectivity and the charge-carrier dynamics, we performed steady-state photoluminescence (PL) and time-resolved photoluminescence (TRPL) measurements under two distinct excitation geometries: front-side (through the perovskite film surface) and rear-side (through the glass substrate). The results are presented in Figure 2, and the corresponding fitting parameters are summarized in Table S6. Under front-side excitation, which primarily probes the top surface and near-surface regions, the control film exhibited a weak PL peak centered at ~655 nm. After dual-surface passivation, all treated films showed a dramatic increase in PL intensity, evidencing effective passivation of non-radiative centers at the perovskite surface and grain boundaries [31]. Notably, the ThMI-based films (PEAI/ThMI and ThMI/ThMI) displayed the highest PL intensities (Figure 2a). Meanwhile, a clear blueshift of the emission peak was observed: the PEAI/ThMI and ThMI/ThMI films shifted to ~648 nm and ~649 nm, respectively. This blueshift suggests a reduction in shallow trap-state density and decreased electronic disorder at the surface [32]. To assess charge extraction at the perovskite/PCBM interface, we measured PL on films with a PCBM top layer under front-side excitation (Figure 2b). Deposition of PCBM substantially quenched the PL for all films, indicating efficient electron extraction [33]. The quenching was most pronounced for the PEAI/ThMI and ThMI/ThMI films, consistent with their superior passivation and interfacial charge transfer.

To further explore charge transport at the buried NiO_x_/perovskite interface, we conducted rear-side excited PL measurements (Figure 2c). Compared to the control film, both PEAI/ThMI and ThMI/ThMI samples exhibit markedly quenched emission, with PEAI/ThMI showing the weakest intensity and the most pronounced blue shift. Since rear-side excitation predominantly generates carriers near the buried interface, the observed PL quenching in the modified films signifies rapid hole extraction at this interface. The more efficient quenching in PEAI/ThMI indicates a superior hole-extraction pathway, which is consistent with the enhanced FF of the corresponding devices.

The TRPL decay curves under front-side excitation (without PCBM) are shown in Figure 2d and Table S6. The average carrier lifetime (τ_ave_) increased markedly from 8.09 ns for the control to 9.51 ns (PEAI/PEAI), 12.11 ns (PEAI/ThMI), 11.82 ns (ThMI/ThMI), and 10.39 ns (ThMI/PEAI). The prolonged lifetimes in passivated films confirm effective suppression of trap-assisted recombination, which is critical for achieving high V_oc_ [34]. When PCBM was deposited (front-side excitation, Figure 2e), all films showed accelerated decays due to electron extraction. The lifetimes were substantially shortened, with the control/PCBM exhibiting τ_ave_ = 5.27 ns and the PEAI/ThMI/PCBM showing the shortest lifetime of 3.30 ns. This efficient quenching demonstrates that the passivation layers not only reduce surface recombination but also facilitate superior charge transfer to the ETL. Under rear-side excitation (Figure 2f), the control film had a τ_ave_ of 5.41 ns. Interestingly, the passivated films displayed shorter lifetimes: PEAI/PEAI (4.27 ns), PEAI/ThMI (2.79 ns), and ThMI/ThMI (3.16 ns). While a longer lifetime would typically indicate reduced bulk recombination, the shorter lifetimes observed here may be attributed to more efficient carrier extraction from the bottom interface. Notably, the PEAI/ThMI combination again exhibited the most pronounced effect, suggesting a synergistic interplay where ThMI passivates top-surface defects and PEAI beneficially interacts with the buried NiO_x_ interface, thereby reducing non-radiative losses throughout the entire film [30]. These results demonstrate that the dual passivation strategy, particularly the PEAI/ThMI combination, effectively suppresses non-radiative recombination across the entire film and at both interfaces, leading to superior charge-carrier dynamics and device performance.

**Figure 3:**
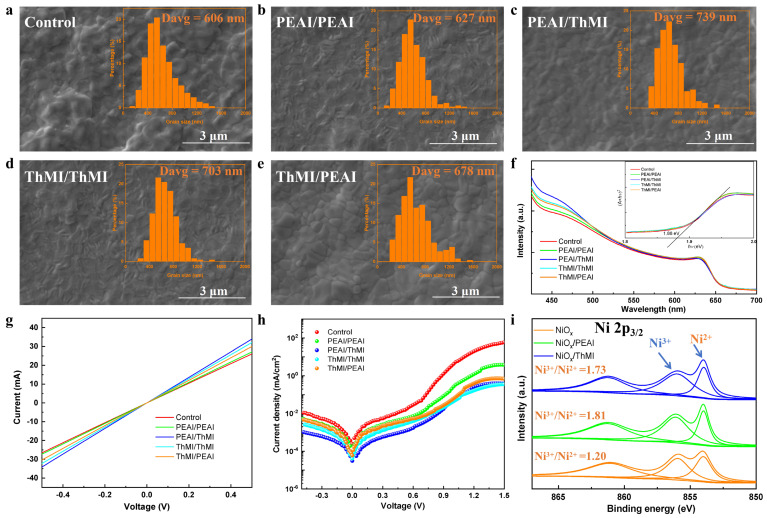
Top−view SEM images of (**a**) control, (**b**) PEAI/PEAI, (**c**) PEAI/ThMI, (**d**) ThMI/ThMI, and (**e**) ThMI/PEAI perovskite films. (**f**) UV–vis absorption spectra. (**g**) Conductivity derived from dark I–V curves of the control and dual-interface-passivated devices. (**h**) Dark J−V curves of the same devices. (**i**) Ni 2p XPS spectra of pristine NiO_x_, NiO_x_/PEAI, and NiO_x_/ThMI films.

To systematically investigate the impact of dual-interface passivation on the quality of CsPbI_2_Br perovskite films, top-view scanning electron microscopy (SEM) was performed. As depicted in Figure 3a, the pristine CsPbI_2_Br film presents a relatively rough surface with obvious pinholes. These morphological defects imply abundant structural imperfections, which act as efficient non-radiative recombination centers [35]. In contrast, the films modified with PEAI and ThMI at both interfaces (Figure 3b–e) present a significantly more uniform, compact, and pinhole-free morphology. A notable increase in average grain size is observed, ascending from ~606 nm for the control to ~739 nm for the PEAI/ThMI-treated film. Such evolution is ascribed to inhibited heterogeneous nucleation and accelerated growth of larger, well-ordered crystalline grains [30].

The optical properties of the perovskite films were evaluated via UV–vis absorption spectroscopy (Figure 3f). All films display analogous absorption profiles with an absorption onset at ~659 nm, corresponding to a consistent optical bandgap of ~1.88 eV, which aligns with the values reported for high-quality CsPbI_2_Br [28,36]. However, a discernible enhancement in absorption intensity across the visible spectrum is observed for the treated films, particularly for the PEAI/ThMI and ThMI/ThMI configurations. This intensified light-harvesting capability is primarily attributed to the superior film crystallinity, reduced defect density, and enhanced light scattering from larger grains, which minimize parasitic absorption losses [37]. The absence of characteristic 2D absorption peaks around 500 nm confirms that passivation with organic ammonium salts leads to surface cation termination on CsPbI_2_Br, rather than forming a 2D capping layer. To quantify the structural disorder of the films, we extracted the Urbach energy (E_u_) from the exponential absorption edge of the spectra in Figure 3f. The E_u_ values, obtained by fitting ln(A) vs. photon energy in the sub-bandgap region (1.85–1.95 eV), are 45.2 meV (control), 39.8 meV (PEAI/PEAI), 32.5 meV (PEAI/ThMI), 34.1 meV (ThMI/ThMI), and 36.7 meV (ThMI/PEAI). The lowest E_u_ for the PEAI/ThMI sample indicates the most ordered lattice and the fewest tail states, in excellent agreement with its lowest trap density (3.05 × 10^15^ cm^−3^) from SCLC and its longest carrier lifetime from TRPL. Notably, the asymmetric dual-passivation outperforms both symmetric configurations, confirming that PEAI at the bottom and ThMI at the top synergistically reduce interfacial disorder. These results directly correlate with the enhanced V_oc_ and FF of the PEAI/ThMI device, further substantiating that effective suppression of sub-bandgap states is key to the improved photovoltaic performance. Figure 3g presents the conductivity derived from dark J–V measurements. The dual-passivated devices, especially the PEAI/ThMI-based one, demonstrate markedly higher conductivity compared to the control. Meanwhile, the dark J–V curves in Figure 3h reveal that the passivated devices exhibit a substantially lower leakage current under reverse bias. The reduction in leakage current and enhancement in conductivity indicate a more favorable energy-level alignment and a reduction in trap-assisted recombination at both the NiO_x_/perovskite and perovskite/PCBM interfaces [27].

To clarify the chemical passivation mechanism at the buried NiO_x_/perovskite interface, X-ray photoelectron spectroscopy (XPS) was performed on the Ni 2p core level of pristine and modified NiO_x_ films (Figure 3i). The Ni 2p_3/2_ spectrum is deconvoluted into two primary components: Ni^2+^ (≈853.99 eV) and Ni^3+^ (≈855.93 eV) species. The presence of Ni^3+^ is intrinsically linked to nickel vacancies, which, while contributing to p-type conductivity, can also act as degradation hotspots by catalyzing interfacial redox reactions [38]. Quantitative analysis reveals that the Ni^3+^/Ni^2+^ ratio increases remarkably from 1.20 for pristine NiO_x_ to 1.81 and 1.73 after treatment with PEAI and ThMI, respectively. This significant increase is ascribed to a strong coordination interaction between the ammonium cations and the NiO_x_ surface, which draws electron density away from the Ni cations, thereby stabilizing the higher oxidation state. The resultant augmentation of p-type character in the hole transport layer is beneficial for enhancing hole extraction efficiency and reducing interfacial recombination losses, which directly implies improved energy-level alignment at the NiO_x_/perovskite interface, ultimately contributing to the superior photovoltaic performance of the passivated devices [15].

**Figure 4:**
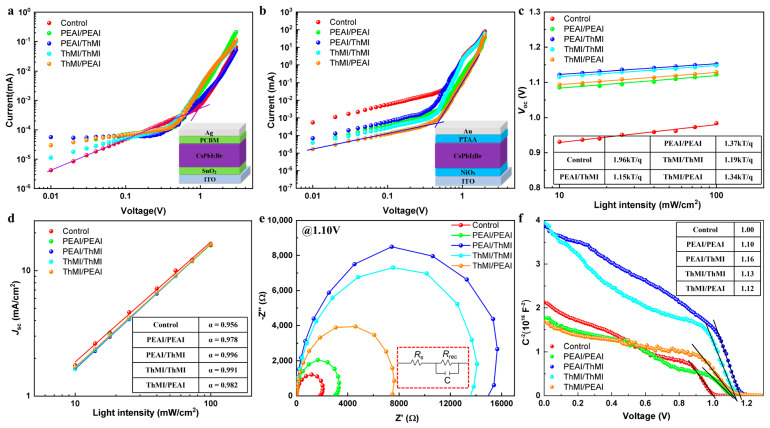
Dark I−V curves of (**a**) electron−only devices (ITO/SnO_2_/CsPbI_2_Br/PCBM/Ag) and (**b**) hole−only devices (ITO/NiO_x_/CsPbI_2_Br/PTAA/Au). Light−intensity dependence of (**c**) V_oc_ and (**d**) J_sc_ for the control and dual−interface−passivated devices. (**e**) Nyquist plots measured in the dark at a forward bias of 1.10 V. (**f**) Mott−Schottky analysis of the same devices.

To explain the origin of the enhanced photovoltaic performance, we systematically investigated the charge carrier dynamics and recombination mechanisms using space-charge-limited current (SCLC), light-intensity-dependent measurements, electrochemical impedance spectroscopy (EIS), and Mott–Schottky analysis. SCLC measurements were performed on electron-only devices (ITO/SnO_2_/CsPbI_2_Br/PCBM/Ag) and hole-only devices (ITO/NiO_x_/CsPbI_2_Br/PTAA/Au) under dark conditions to quantify the trap-state density (*N_t_*) (Figure 4a,b). The trap-filled limit voltage (*V_TFL_*) is directly correlated to the trap density through the equation [39]:

Nt=2εε0VTFLeL2

where *ε* is the relative dielectric constant (≈8.5 for CsPbI_2_Br) [40], *ε*_0_ is the vacuum permittivity, *e* is the elementary charge, and *L* is the film thickness. For electron-only devices, *V_TFL_* decreased substantially from 0.931 V (control, *N_t_* = 7.92 × 10^15^ cm^−3^) to 0.358 V for PEAI/ThMI (*N_t_* = 3.05 × 10^15^ cm^−3^) and 0.399 V for ThMI/ThMI (*N_t_* = 3.39 × 10^15^ cm^−3^) (Table S7). Similarly, for hole-only devices, *V_TFL_* decreased from 0.594 V (control) to 0.378 V for PEAI/ThMI and 0.419 V for ThMI/ThMI, corresponding to hole trap density reductions from 5.06 × 10^15^ cm^−3^ to 3.22 × 10^15^ cm^−3^ and 3.57 × 10^15^ cm^−3^, respectively. The marked reduction in both electron and hole trap densities upon bifacial passivation confirms effective defect passivation at both the buried NiO_x_/perovskite interface and the top perovskite/PCBM interface, consistent with the enhanced photoluminescence and improved V_oc_ [27].

The dependence of V_oc_ and J_sc_ on incident light intensity (P_light_) was analyzed to probe the recombination pathways (Figure 4c,d). The relationship Voc=nkTlnPlightq+c V allows extraction of the ideality factor (n), where *n* = 1 indicates dominant bimolecular radiative recombination while *n* ≈ 2 suggests significant trap-assisted Shockley–Read–Hall (SRH) recombination [41]. The control device exhibited a large *n* of 1.96, indicative of severe trap-mediated non-radiative losses. In contrast, the *n* values for PEAI/ThMI and ThMI/ThMI devices were dramatically reduced to 1.15 and 1.19, respectively, signifying effective suppression of trap-assisted recombination pathways. This substantial reduction correlates directly with the increased V_oc_ and FF of the modified devices [35,42]. The power-law relationship $J_{sc}\propto {P_{light}}^{\alpha }$ was employed to evaluate charge extraction efficiency, where α close to 1 denotes minimal carrier losses before extraction [43]. The fitted α values increased from 0.956 for the control to 0.996 for PEAI/ThMI and 0.991 for ThMI/ThMI, confirming that the dual passivation strategy facilitates more efficient charge sweep-out and mitigates carrier accumulation at the interfaces.

EIS was employed to investigate interfacial charge transfer and recombination dynamics. Nyquist plots measured at a forward bias of 1.10 V under dark conditions are displayed in Figure 4e, with data fitted using the equivalent circuit model shown in the inset. The intermediate-frequency semicircle corresponds to the recombination resistance (R_rec_), which inversely correlates with the recombination rate [44]. The control device exhibited an R_rec_ of 1829 Ω, which increased substantially to 14,984 Ω for the PEAI/ThMI device and 13,581 Ω for the ThMI/ThMI device. The significantly enlarged R_rec_ for the modified devices directly evidences substantial suppression of charge recombination, stemming from effective passivation of defect states at both interfaces and consequent reduction in non-radiative loss channels. This observation is in excellent agreement with the reduced n values and prolonged carrier lifetimes obtained from TRPL. Mott–-Schottky analysis was performed to evaluate the built-in potential (V_bi_), which provides the driving force for charge carrier separation and collection [45,46]. Figure 4f presents the C^−2^–V characteristics measured in the dark. The V_bi_ was determined from the intercept of the linear extrapolation of the C^−2^ curve with the voltage axis. The control device exhibited a V_bi_ of 1.00 V. Upon dual interface passivation, V_bi_ increased to 1.16 V for PEAI/ThMI and 1.13 V for ThMI/ThMI. The enhanced V_bi_ indicates a stronger internal electric field across the perovskite absorber, which promotes more efficient drift-assisted charge extraction and reduces the probability of carrier trapping and recombination. The larger V_bi_ in the PEAI/ThMI device is consistent with its superior V_oc_ and FF. As evidenced by the significantly increased recombination resistance and built-in potential, the PEAI/ThMI dual passivation establishes a favorable energy-level alignment that enhances charge extraction and reduces trap-assisted recombination losses.

**Figure 5:**
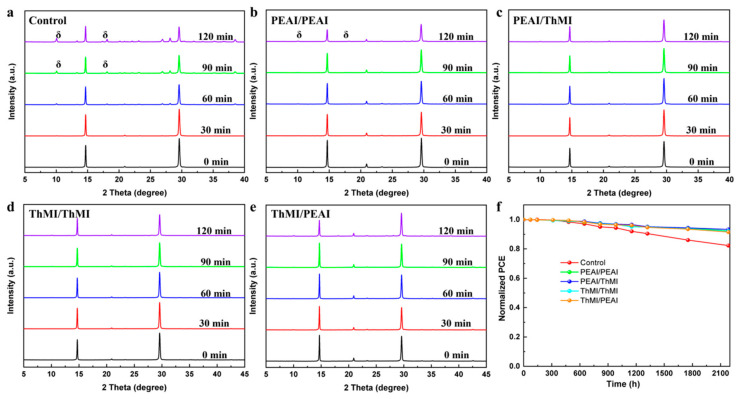
XRD patterns of the perovskite films: (**a**) control, (**b**) PEAI/PEAI, (**c**) PEAI/ThMI, (**d**) ThMI/ThMI, and (**e**) ThMI/PEAI. (**f**) Stability of unencapsulated CsPbI_2_Br PSC devices with and without passivation, stored in an N_2_-filled glovebox at ambient temperature.

To evaluate the structural integrity of the perovskite films, X-ray diffraction (XRD) patterns of the control and modified films were compared before and after aging. As shown in Figure 5a–e, the fresh control film exhibits a strong (100) diffraction peak at ~14.5°, characteristic of the photoactive α-phase CsPbI_2_Br [47,48]. Upon aging, the control film shows the emergence of a distinct non-perovskite δ-phase peak, confirming its structural degradation [49]. In contrast, the PEAI/ThMI (Figure 5c) and ThMI/ThMI (Figure 5d) films demonstrate remarkable phase stability. Their characteristic perovskite peaks remain intense and sharp, with a negligible appearance of the δ-phase peak. This enhanced structural integrity is attributed to the synergistic passivation effect of the organic ammonium salts, which effectively chelate undercoordinated Pb^2+^ ions and fill halide vacancies [24,27]. This surface passivation reduces the nucleation sites for degradation, immobilizes surface halide ions, and suppresses ion migration, thereby mitigating lattice distortion and phase transition [50,51,52]. The impact of this improved stability on device lifetime was assessed by monitoring the PCE of unencapsulated devices stored in an N_2_-filled glovebox at ambient temperature (Figure 5f). The control device exhibited a rapid decline, retaining only ~82% of its initial PCE after 2100 h. In comparison, the ThMI/ThMI and PEAI/ThMI devices showed significantly enhanced long-term stability, retaining ~92% and ~93% of their initial efficiencies, respectively, under identical conditions. For unencapsulated devices stored in ambient air, after 1200 min of storage, the PEAI/ThMI device retains 80% of its initial PCE, whereas the control device maintains only 63% of its original efficiency (Figures S10–S13). This superior operational stability underscores the critical role of dual-interface passivation in protecting the perovskite lattice and ensuring the long-term durability of the devices.

## 3. Conclusions

In summary, we have elucidated the different passivation functions of PEAI and ThMI at the bottom and top interfaces of inverted CsPbI_2_Br PSCs. Bottom modification with PEAI primarily enhances the FF by improving interfacial contact, whereas top passivation with ThMI boosts the V_oc_ by neutralizing undercoordinated Pb^2+^ defects via its thiophene moiety. Exploiting this complementary selectivity, an asymmetric dual-interface architecture (PEAI at the bottom, ThMI at the top) synergistically optimizes hole extraction and suppresses non-radiative recombination, yielding a champion efficiency of 15.44% (V_oc_ = 1.15 V, FF = 82.15%) and retaining over 93% of initial performance after 2100 h.

## Figures and Tables

**Table 1:** Photovoltaic parameters of champion CsPbI_2_Br PSCs based on control, PEAI/PEAI, PEAI/ThMI, ThMI/ThMI, and ThMI/PEAI devices (Bottom/Top).

Devices (Bottom/Top)	Scan Direction	V_oc_ (V)	Measured J_sc_ (mA/cm^2^)	Integrated J_sc_ (mA/cm^2^)	FF (%)	PCE (%)	HI (%)
Control	Reverse	0.98	15.94	14.82	75.49	11.79	9.84
	Forward	0.93	15.90		71.92	10.63	
PEAI/PEAI	Reverse	1.12	16.25	15.53	80.79	14.70	2.31
	Forward	1.11	16.22		79.74	14.36	
PEAI/ThMI	Reverse	1.15	16.34	15.86	82.15	15.44	1.55
	Forward	1.14	16.32		81.68	15.20	
ThMI/ThMI	Reverse	1.15	16.28	15.74	80.53	15.08	2.78
	Forward	1.13	16.22		80.01	14.66	
ThMI/PEAI	Reverse	1.13	16.30	15.66	80.41	14.81	3.38
	Forward	1.12	16.25		78.65	14.31	

The configuration order is ITO/NiO_x_/Bottom-passivator/CsPbI_2_Br/Top-passivator/PCBM/BCP/Ag. “PEAI/PEAI” means PEAI at both bottom and top interfaces, etc.

## Data Availability

The original contributions presented in this study are included in the article/Supplementary Materials. Further inquiries can be directed to the corresponding authors.

## Supplementary Materials

The following supporting information can be downloaded at https://www.mdpi.com/article/10.3390/nano16130795/s1. The supporting information includes: Table S1: Comparison of recently reported inverted p-i-n CsPbI_2_Br PSCs; Experimental Section; Tables S2–S5: Photovoltaic parameters for top/bottom passivation with PEAI/ThMI at different concentrations; Table S6: TRPL fitting parameters and average lifetimes (Figure 2d–f); Table S7: Trap density from SCLC measurements (hole-only and electron-only devices); Figures S1–S4: Corresponding J–V curves; Figures S5 and S6: O 1s and I 3d XPS spectra of NiO_x_, NiO_x_/PEAI, NiO_x_/ThMI; Figures S7–S13: Normalized V_oc_, J_sc_, and FF vs. storage time for unencapsulated devices.
