# Supplementary material for: Asymmetric Dual-Interface Passivation with Functionalized Ammonium Halides for High-Performance Inverted CsPbI2Br Perovskite Solar Cells
Source: Nanomaterials (Basel). 2026 Jun 27;16(13):795. doi: 10.3390/nano16130795 (PMC13362995; doi:10.3390/nano16130795)
Supplement: Supplementary file 1 [file nanomaterials-16-00795-s001.zip › nanomaterials-4369208-supplementary.pdf]

Asymmetric Dual-Interface Passivation with Functionalized Ammonium Halides for High-Performance Inverted $CsPbI_2Br$ Perovskite Solar Cells

Xin Liu*, Chengguo Liu, Wei Li, Wangyang Song, Xiaoxuan Li, Bo Li, Kun Zhao, Shu Wang, Jie Li, and Dingyu Yang*

Optoelectronic Sensor Devices and Systems Key Laboratory of Sichuan Provincial Universities, Sichuan Meteorological Optoelectronic Sensor Technology and Application Engineering Research Center, Information Materials and Device Applications Key Laboratory of Sichuan Provincial Universities, College of Optoelectronic Engineering (Chengdu IC Valley Industrial College), Chengdu University of Information Technology, Chengdu 610225, China

*Corresponding author, E-mail: come_on_liuxin@163.com, yangdingyu@cuit.edu.cn

Table S1. Comparative photovoltaic performance of recently reported inverted p-i-n $CsPbI_2Br$ perovskite solar cells.

Configuration	PCE (%)	V_{oc} (V)	J_{sc} (mA/cm^2)	FF (%)	Ref.
**ITO/NiO$_x$/PEAI/CsPbI$_2$Br/ThMI/PCBM/BCP/Ag**	**15.44**	**1.15**	**16.34**	**82.15**	**This work**
ITO/NiO$_x$/CsPbI$_2$Br/Ti$_{0.9}$Sn$_{0.1}$O$_2$/Ag	14.00	1.15	15.90	76.60	J. Energy Chem. **2022**, 68, 176
ITO/NiO$_x$/CsPbI$_2$Br/PCBM/BCP/Ag	14.67	1.21	15.99	76.00	Sol. RRL **2022**, 6, 2200690
ITO/NiO$_x$/CsPbI$_2$Br/BP-HI/ZnO@C$_{60}$/Ag	15.36	1.21	16.31	78.20	Chem. Eng. J. **2022**, 435, 134760
ITO/NiO$_x$/CsPbI$_2$Br/PAMAM/ZnO@C$_{60}$/Ag	15.83	1.28	15.80	78.48	ACS Appl. Mater. Interfaces **2023**, 15, 25550
ITO/2PACz/CsPbI$_2$Br/PEACl/ICBA/BCP/Ag	16.00	1.29	15.25	81.50	ACS Energy Lett. **2023**, 8, 2077
FTO/NiO$_x$/CsPbI$_2$Br/TiO$_2$/Al	17.10	1.26	16.40	83.10	Adv. Energy Mater. **2023**, 2301607
ITO/NiO$_x$/CsPbI$_2$Br/Nano-BWO/PCBM/Bphen/Ag	15.71	1.19	16.22	81.64	Chem. Eng. J. **2023**, 466, 143273
ITO/NiO$_x$/CsPbI$_2$Br/ZnO/C$_{60}$/Al	12.17	1.13	14.69	73.00	Sol. RRL **2023**, 2300369
ITO/CsPbI$_2$Br/PCBM/BCP/Ag	12.92	1.14	15.03	75.33	Appl. Phys. Lett. **2024**, 125, 243902
FTO/NiO$_x$/CsPbI$_2$Br/C$_{60}$/BCP/Ag	8.94	1.04	14.42	59.8	ChemSusChem **2024**, 17, e202301722
ITO/NiO$_x$/CsPbI$_2$Br/CFPMAI/PCBM/BCP/Ag	14.43	1.12	16.31	79.02	J. Chem. Phys. **2024**, 160, 094705
ITO/NiO$_x$/MeO-2PACz/CsPbI$_2$Br/PEACl/PCBM/BCP/Cu	13.21	1.148	15.60	73.70	Adv. Funct. Mater. **2025**, 35, e02970
ITO/NiO$_x$/MeO-2PACz/CsPbI$_2$Br+sulfolane/PCBM/BCP/Ag	12.16	1.12	14.56	74.54	ACS Appl. Energy Mater. **2026**, 9, 542

Experimental Section

Materials: Lead iodide (PbI_2, 99.9985%), lead bromide ($PbBr_2$, 99.999%), and nickel oxide (NiO_x, 99.999%) were purchased from Advanced Election Technology (China). Cesium iodide (CsI, 99.999%), poly[bis(4-phenyl)(2,4,6-trimethylphenyl)amine] (PTAA), [6,6]-phenyl-C_{61}-butyric acid methyl ester (PCBM), and bathocuproine (BCP) were obtained from Xi'an Yuri Solar Co., Ltd. Phenylethylammonium iodide (PEAI) and 2-thiophenemethylammonium iodide (ThMI) were purchased from commercial sources. Anhydrous isopropanol (IPA, 99.5%), anhydrous dimethyl sulfoxide (DMSO, 99.9%), anhydrous N,N-dimethylformamide (DMF, 99.8%), and anhydrous chlorobenzene (CB, 99.8%) were acquired from Sigma-Aldrich. A tin(IV) oxide (SnO_2) colloid precursor was ordered from Alfa Aesar, and evaporation materials (Au, 99.99%; Ag, 99.999%) were procured from ZhongNuo Advanced Material (Beijing) Technology Co., Ltd. All chemicals and solvents were used as received without further purification.

Solar cell fabrication: Patterned indium-tin-oxide (ITO)-coated glass substrates (20 mm × 20 mm) were ultrasonically cleaned sequentially with diluted detergent, deionized water, acetone, and isopropanol for 20 min each. After drying under a N_2 stream, the substrates were treated with UV-ozone for 20 min. A NiO_x nanocrystal suspension (20 mg/mL in deionized water) was spin-coated onto the ITO substrates at 2500 rpm for 30 s and annealed in air at 150 °C for 30 min to form the hole transport layer (HTL). For bottom-interface passivation, PEAI or ThMI solutions in IPA

(0.5-1.5 mg/mL) were spin-coated on the NiO_x films at 3000 rpm for 30 s, followed by annealing at 100 °C for 5 min. The substrates were then transferred into a N_2-filled glovebox. The $CsPbI_2Br$ precursor solution was prepared by dissolving 277 mg PbI_2 (0.6 M), 312 mg CsI (1.2 M), and 220 mg $PbBr_2$ (0.6 M) in 1 mL of anhydrous DMSO, stirred at 60 °C for 2 h, and filtered through a 0.22 μm polytetrafluoroethylene (PTFE) filter. The perovskite film was deposited by spin-coating the precursor at 2000 rpm for 120 s, followed by sequential annealing at 50 °C for 2 min and then at 150 °C for 10 min. For top-interface passivation, PEAI or ThMI solutions in IPA (0.5-1.5 mg/mL) were spin-coated onto the perovskite film at 3000 rpm for 30 s and annealed at 100 °C for 5 min. Subsequently, PCBM (20 mg/mL in CB) and BCP (0.5 mg/mL in IPA) were sequentially spin-coated at 2000 rpm for 30 s and 6000 rpm for 30 s, respectively. Finally, a 100 nm Ag electrode was thermally evaporated through a shadow mask under a base pressure of $\approx 8\times10^{-6}$ Torr at a deposition rate of ≈ 5 Å/s, defining an active area of 0.048 cm^2.

Characterization and measurements: The current density-voltage (J-V) characteristics were measured under AM 1.5G illumination (100 mW/cm^2) using an AAA solar simulator (Sirius-SS150A-D) calibrated with a Konica-Minolta AK-200 reference cell. The external quantum efficiency (EQE) spectra were recorded in ambient air using an Enlitech QE-R system. Steady-state photoluminescence (PL) and time-resolved photoluminescence (TRPL) spectra were acquired on a PicoQuant FluoTime 300 spectrometer equipped with a 510 nm picosecond laser (repetition rate 0.2-40 MHz). The PL decays were fitted biexponentially, and the average carrier

lifetimes (τ_{ave}) were calculated using the formula $\tau_{ave} = (A_1\tau_1^2 + A_2\tau_2^2)/(A_1\tau_1 + A_2\tau_2)$. Top-view scanning electron microscopy (SEM) images were taken with a Phenom ProX microscope at 5 kV. X-ray diffraction (XRD) patterns were collected on a Shimadzu XRD-6100 diffractometer using Cu Kα radiation. UV-vis absorption spectra were recorded on a SHIMADZU UV-2600 spectrometer. X-ray photoelectron spectroscopy (XPS) was performed on a PHI Versaprobe 4 system with monochromatic Al Kα radiation. Space-charge-limited current (SCLC) measurements were carried out in the dark using a Keithley 2400 SourceMeter on electron-only devices (ITO/SnO_2/$CsPbI_2Br$/PCBM/Ag) and hole-only devices (ITO/NiO_x/$CsPbI_2Br$/PTAA/Au). The trap density (N_t) was calculated using the formula $N_t = (2\varepsilon\varepsilon_0 V_{TFL})/(eL^2)$, where ε is the relative dielectric constant (≈ 8.5 for $CsPbI_2Br$), ε_0 is the vacuum permittivity, e is the elementary charge, L is the perovskite film thickness, and V_{TFL} is the trap-filled limit voltage. Electrochemical impedance spectroscopy (EIS) and Mott-Schottky analysis were performed in the dark using a CHI 660E electrochemical workstation at a frequency range of 1 Hz to 1 MHz and an AC amplitude of 10 mV. The long-term stability of unencapsulated devices was evaluated by storing them in a N_2-filled glovebox at ambient temperature and measuring the photovoltaic parameters.

Table S2. Photovoltaic parameters of $CsPbI_2Br$ PSCs with top-surface passivation by PEAI at different concentrations, measured under AM 1.5G illumination (100 mW/cm^2).

Devices	V_{oc} (V)	J_{sc} (mA/cm^2)	FF (%)	PCE (%)
PEAI-0.5	1.10	16.21	78.39	13.98
PEAI-1.0	1.12	16.24	78.43	14.27
PEAI-1.5	1.11	16.22	78.28	14.09

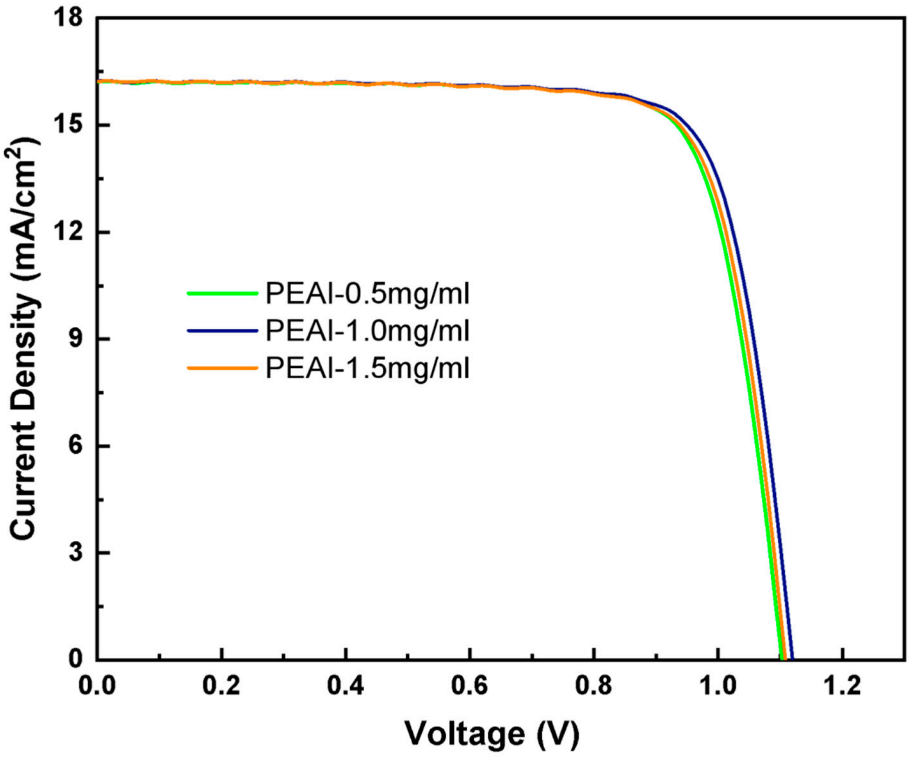

Figure S1. J-V curves of $CsPbI_2Br$ PSCs with top-surface passivation by PEAI at different concentrations, recorded under AM 1.5G illumination (100 mW/cm^2).

Table S3. Photovoltaic parameters of $CsPbI_2Br$ PSCs with top-surface passivation by ThMI at different concentrations, measured under AM 1.5G illumination (100 mW/cm^2).

Devices	V_{oc} (V)	J_{sc} (mA/cm^2)	FF (%)	PCE (%)
ThMI-0.5	1.13	16.38	77.40	14.33
ThMI-1.0	1.15	16.33	78.61	14.76
ThMI-1.5	1.15	16.24	76.52	14.29

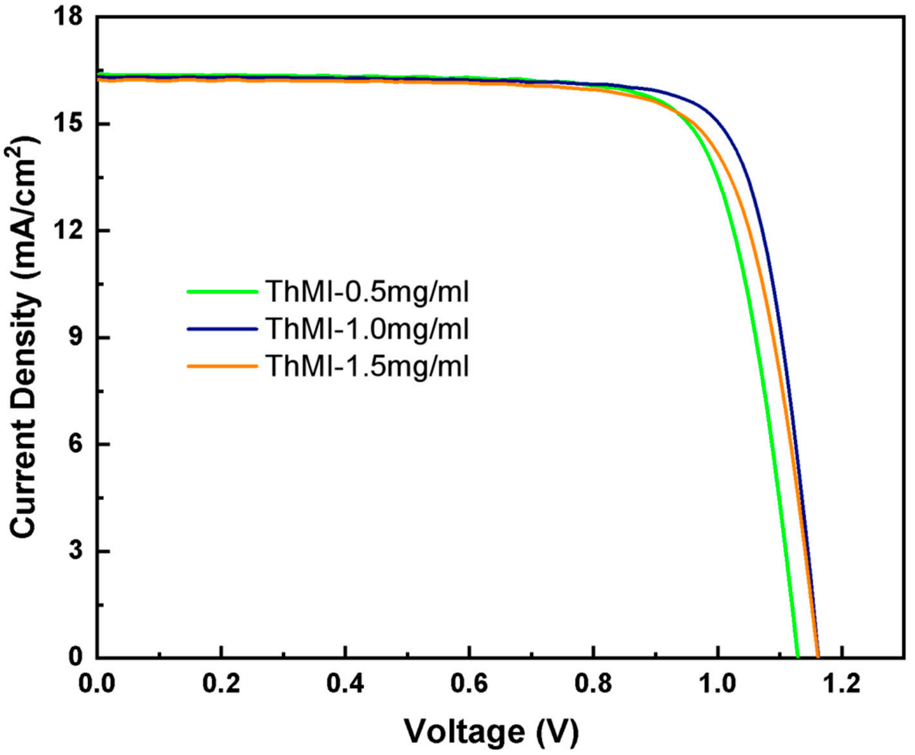

Figure S2. J-V curves of $CsPbI_2Br$ PSCs with top-surface passivation by ThMI at different concentrations, recorded under AM 1.5G illumination (100 mW/cm^2).

Table S4. Photovoltaic parameters of $CsPbI_2Br$ PSCs with bottom-surface passivation by PEAI at different concentrations, measured under AM 1.5G illumination (100 mW/cm^2).

Devices	V_{oc} (V)	J_{sc} (mA/cm^2)	FF (%)	PCE (%)
PEAI-0.5	1.01	16.29	80.51	13.25
PEAI-1.0	1.02	16.28	81.20	13.48
PEAI-1.5	1.02	16.21	80.01	13.23

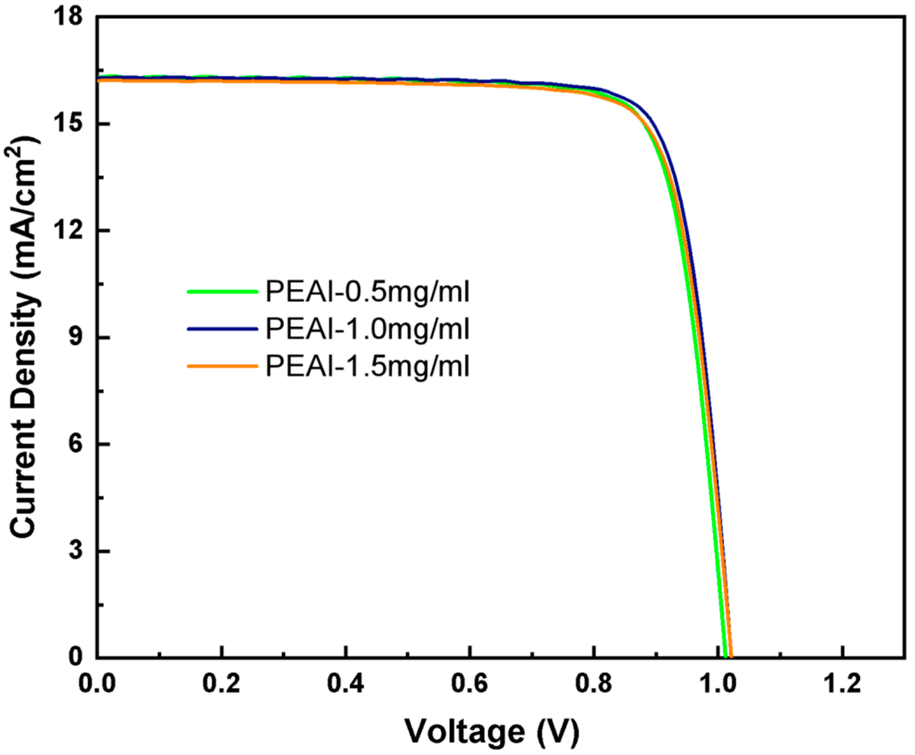

Figure S3. J-V curves of $CsPbI_2Br$ PSCs with bottom-surface passivation by PEAI at different concentrations, recorded under AM 1.5G illumination (100 mW/cm^2).

Table S5. Photovoltaic parameters of $CsPbI_2Br$ PSCs with bottom-surface passivation by ThMI at different concentrations, measured under AM 1.5G illumination (100 mW/cm^2).

Devices	V_{oc} (V)	J_{sc} (mA/cm^2)	FF (%)	PCE (%)
ThMI-0.5	0.98	16.26	80.61	12.85
ThMI-1.0	1.01	16.24	80.76	13.25
ThMI-1.5	1.00	16.22	80.33	13.03

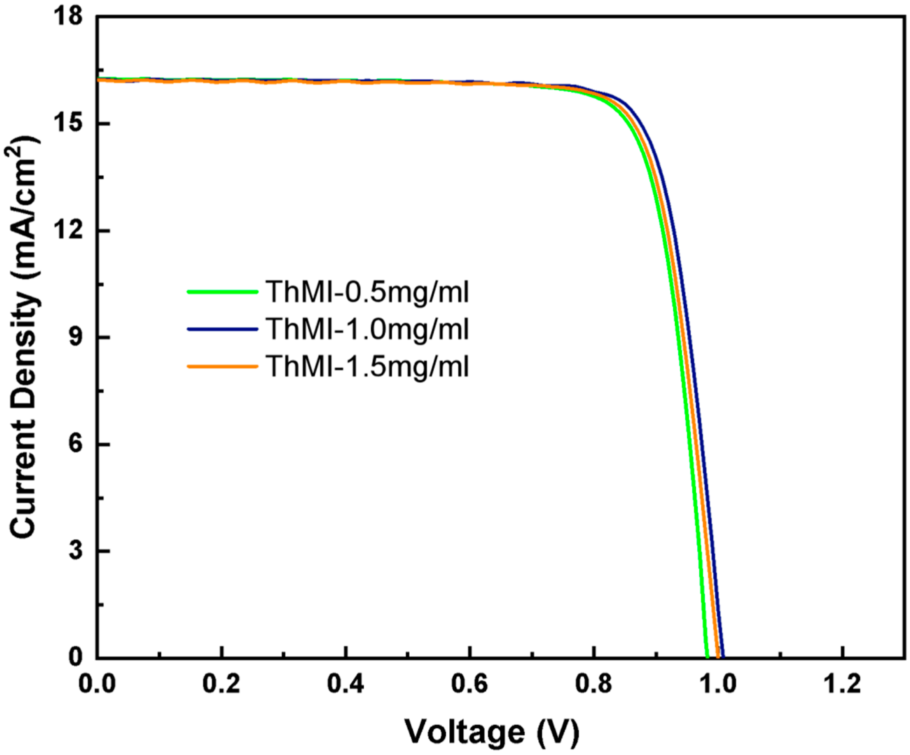

Figure S4. J-V curves of $CsPbI_2Br$ PSCs with bottom-surface passivation by ThMI at different concentrations, recorded under AM 1.5G illumination (100 mW/cm^2).

Table S6. Fitting parameters and average lifetimes derived from the PL decays in Figure 2d-f.

Samples	τ_1 (ns)	A_1 (Cnts)	τ_2 (ns)	A_2 (Cnts)	τ_{ave}(ns)
Control-F	8.4811	6541.2	0.5970	4910	8.0855
PEAI/PEAI-F	10.5443	6364.3	3.752	3214	9.5098
PEAI/ThMI-F	12.6727	7004.0	0.7384	5942	12.1106
ThMI/ThMI-F	12.976	5565.2	3.634	2793	11.8240
ThMI/PEAI-F	10.7644	6764.0	1.096	2695	10.3875
Control/PCBM-F	5.6548	6352.7	0.4662	6157	5.2709
PEAI/PEAI/PCBM-F	4.9162	7504.1	0.3687	7274	4.6080
PEAI/ThMI/PCBM-F	3.5637	7509.7	0.3390	6999	3.3011
ThMI/ThMI/PCBM-F	3.5884	7339.6	0.3567	6524	3.3261
ThMI/PEAI/PCBM-F	4.7053	7265.2	0.4253	6132	4.4019
Control-R	8.720	1289.1	4.6535	10579.6	5.409
PEAI/PEAI-R	9.144	411.3	3.8702	11787.2	4.272
PEAI/ThMI-R	3.7923	3624.2	1.9652	8432	2.7935
ThMI/ThMI-R	3.5850	8723.1	1.8107	5484	3.1574
ThMI/PEAI-R	5.5805	2624.9	2.5428	9276	3.7066

The average carrier lifetime (τ_{ave}) was calculated via the following formula: $\tau_{ave} = (A_1{\tau_1}^2 + A_2{\tau_2}^2)/(A_1\tau_1 + A_2\tau_2)$.

Table S7. Trap-filled limit voltage (V_{TFL}) and trap density (N_t) derived from SCLC measurements of electron-only ($ITO/SnO_2/CsPbI_2Br/PCBM/Ag$) and hole-only ($ITO/NiO_x/CsPbI_2Br/PTAA/Au$) devices fabricated with control, PEAI/PEAI, PEAI/ThMI, ThMI/ThMI, and ThMI/PEAI perovskite films.

Condition	Device structure	V_{TFL} (V)	Trap density (cm^{-3})
Control	$ITO/SnO_2/CsPbI_2Br/PCBM/Ag$	0.931	7.92×10^{15}
PEAI/PEAI		0.507	4.31×10^{15}
PEAI/ThMI		0.358	3.05×10^{15}
ThMI/ThMI		0.399	3.39×10^{15}
ThMI/PEAI		0.455	3.87×10^{15}
Control	$ITO/NiO_x/CsPbI_2Br/PTAA/Au$	0.594	5.06×10^{15}
PEAI/PEAI		0.504	4.29×10^{15}
PEAI/ThMI		0.378	3.22×10^{15}
ThMI/ThMI		0.419	3.57×10^{15}
ThMI/PEAI		0.473	4.03×10^{15}

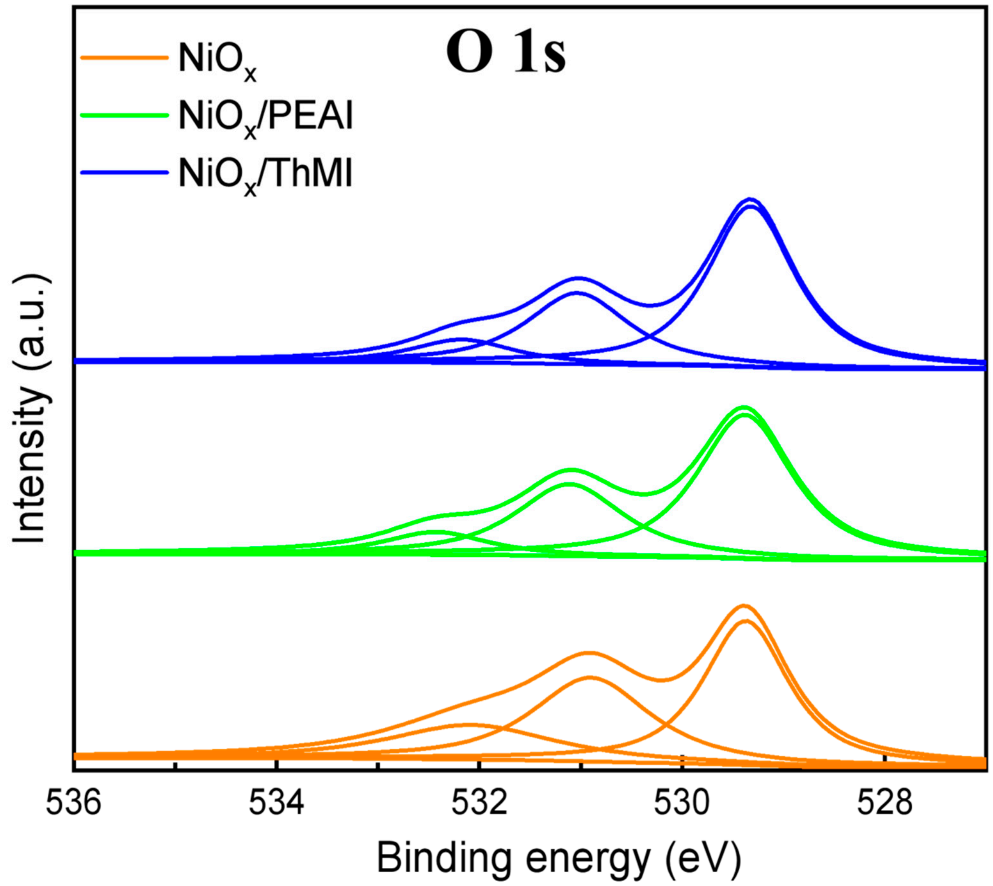

Figure S5. O 1s XPS spectra of NiO_x, NiO_x/PEAI, and NiO_x/ThMI films.

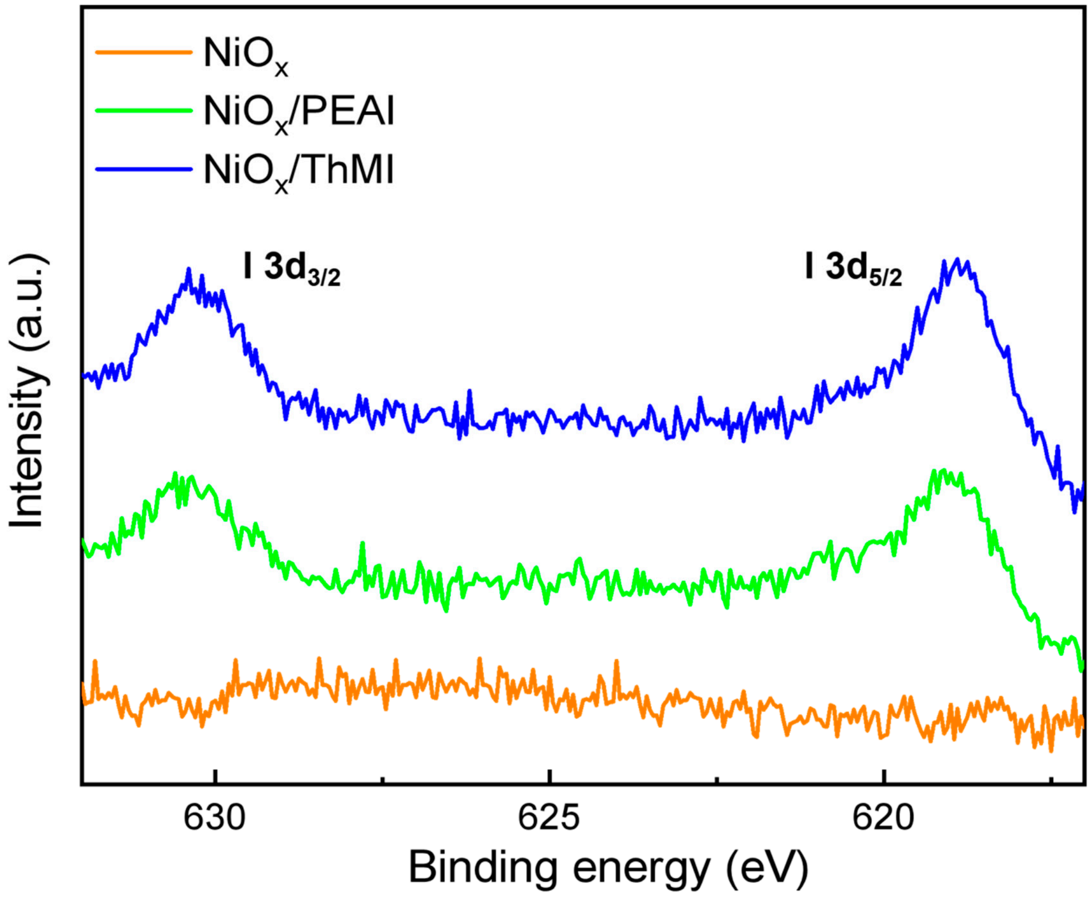

Figure S6. I 3d XPS spectra of NiO_x, NiO_x/PEAI, and NiO_x/ThMI films.

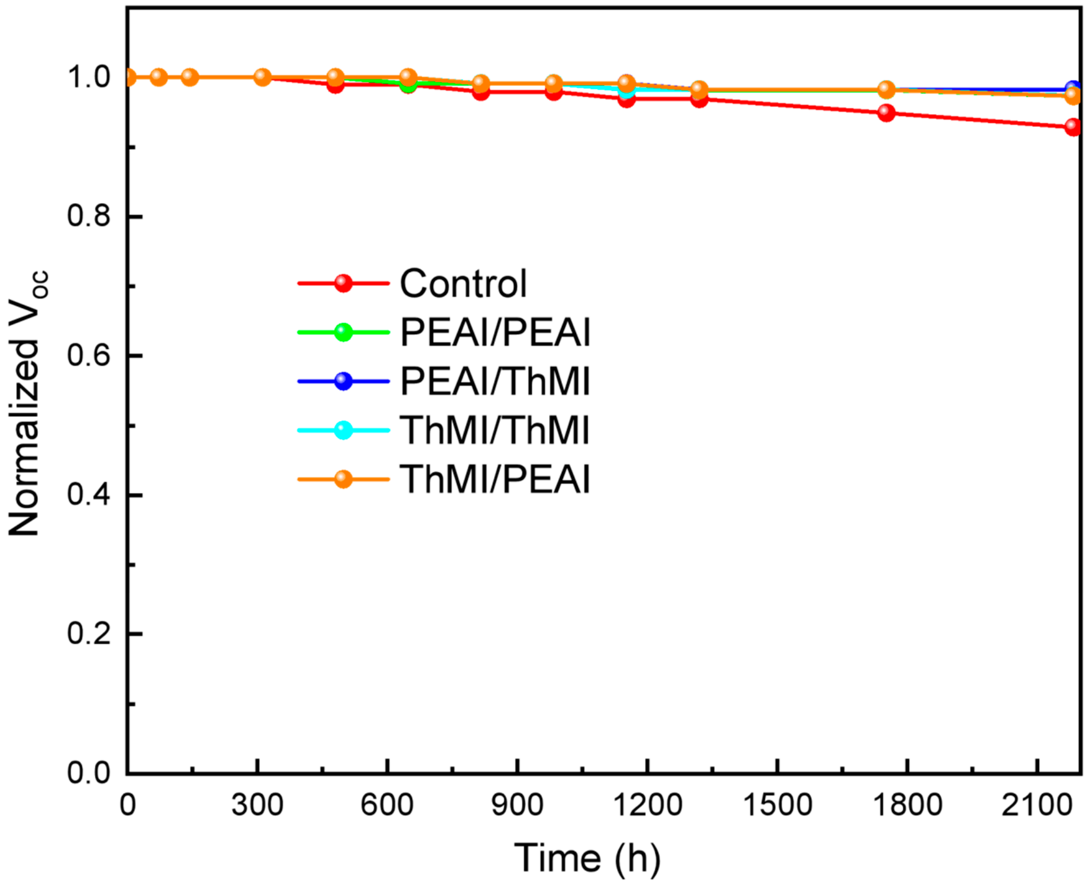

Figure S7. Normalized V_{oc} versus storage time for unencapsulated devices (control, PEAI/PEAI, PEAI/ThMI, ThMI/ThMI, and ThMI/PEAI) stored in an N_2-filled glovebox at ambient temperature.

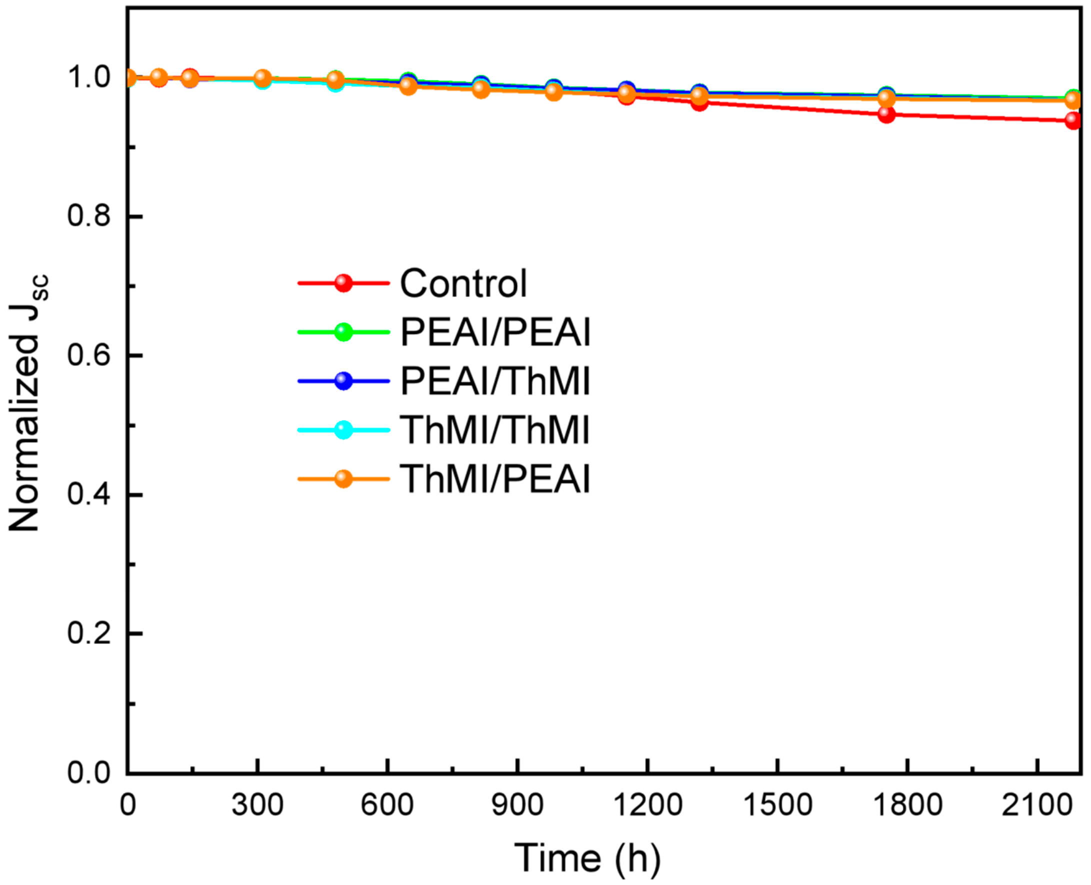

Figure S8. Normalized J_{sc} versus storage time for unencapsulated devices (control, PEAI/PEAI, PEAI/ThMI, ThMI/ThMI, and ThMI/PEAI) stored in an N_2-filled glovebox at ambient temperature.

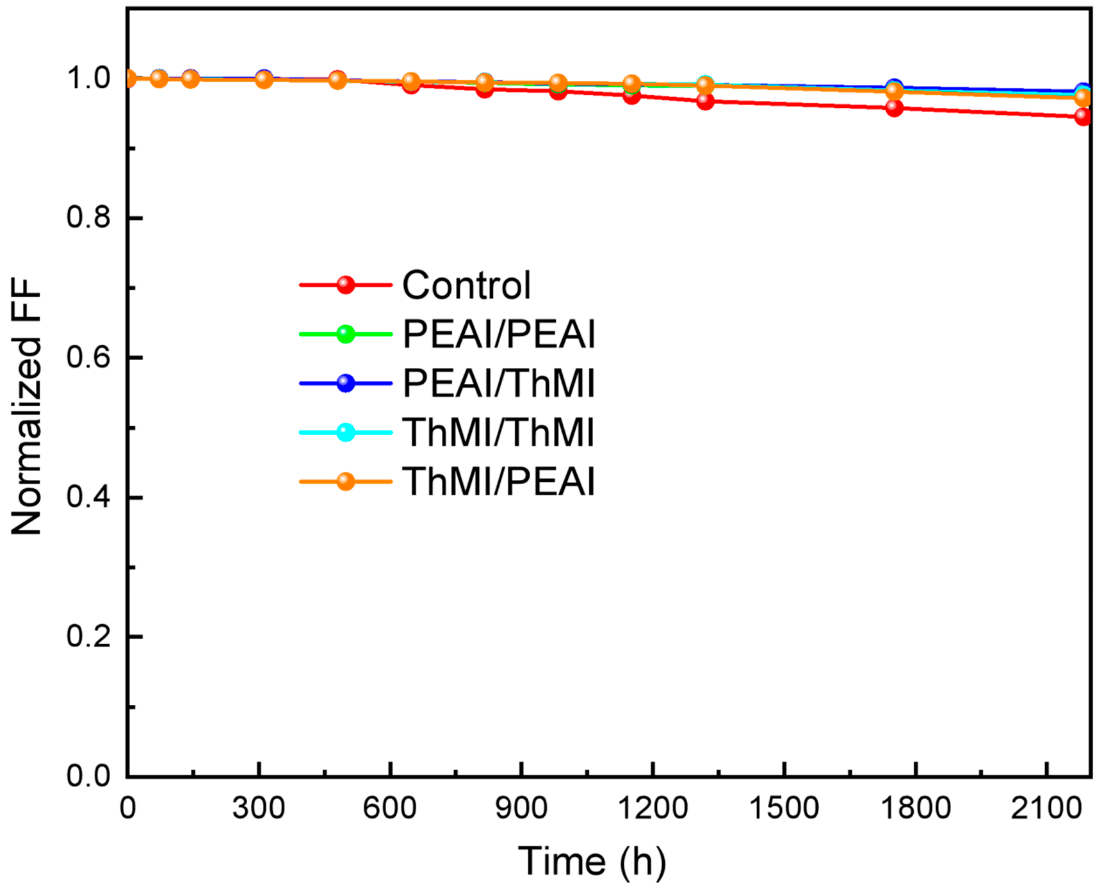

Figure S9. Normalized FF versus storage time for unencapsulated devices (control, PEAI/PEAI, PEAI/ThMI, ThMI/ThMI, and ThMI/PEAI) stored in an N_2-filled glovebox at ambient temperature.

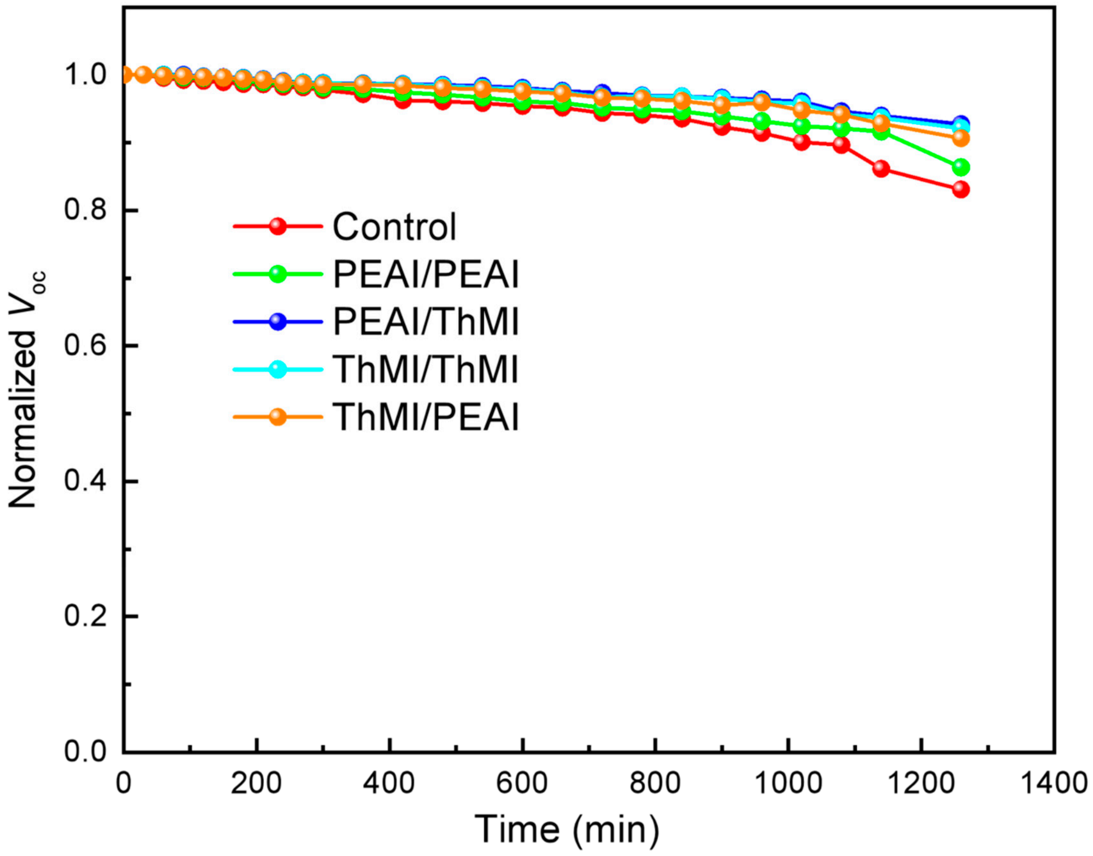

Figure S10. Normalized V_{oc} over time for unencapsulated control and dual-interface passivation devices stored under ambient air (RH 30-40%) at room temperature.

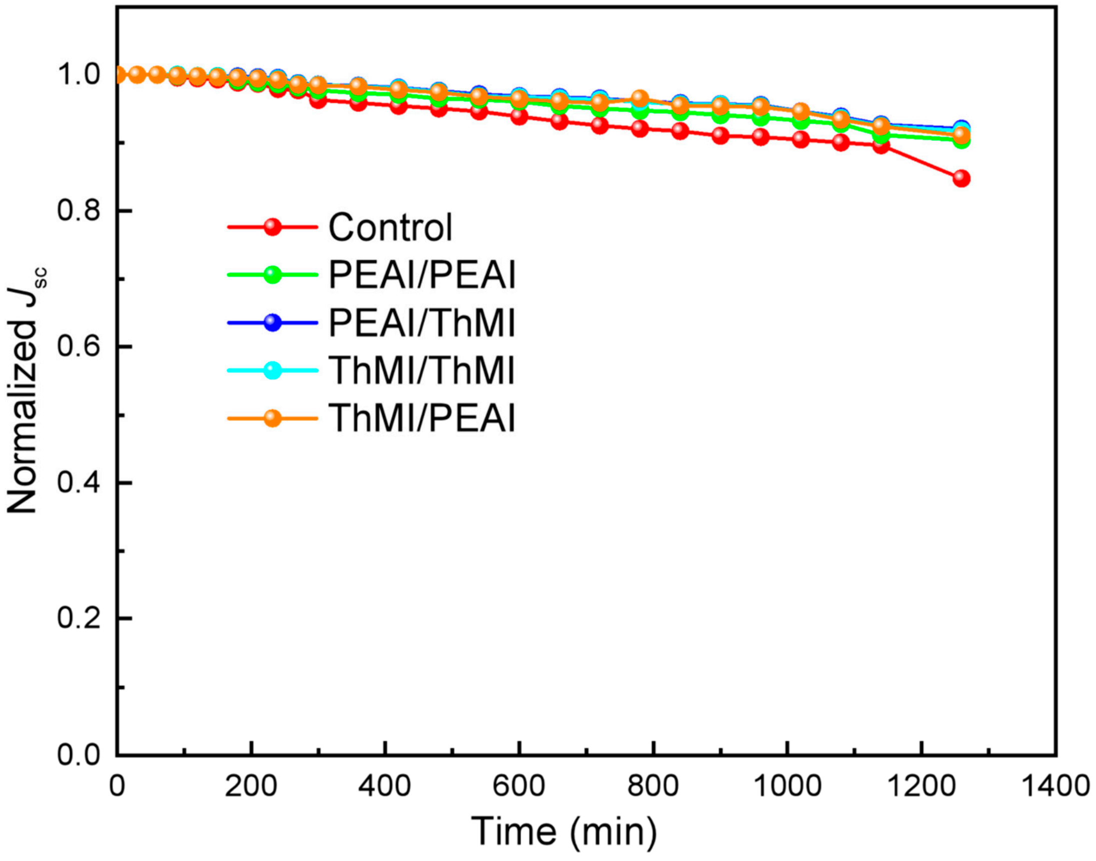

Figure S11. Normalized J_{sc} over time for unencapsulated control and dual-interface passivation devices stored under ambient air (RH 30-40%) at room temperature.

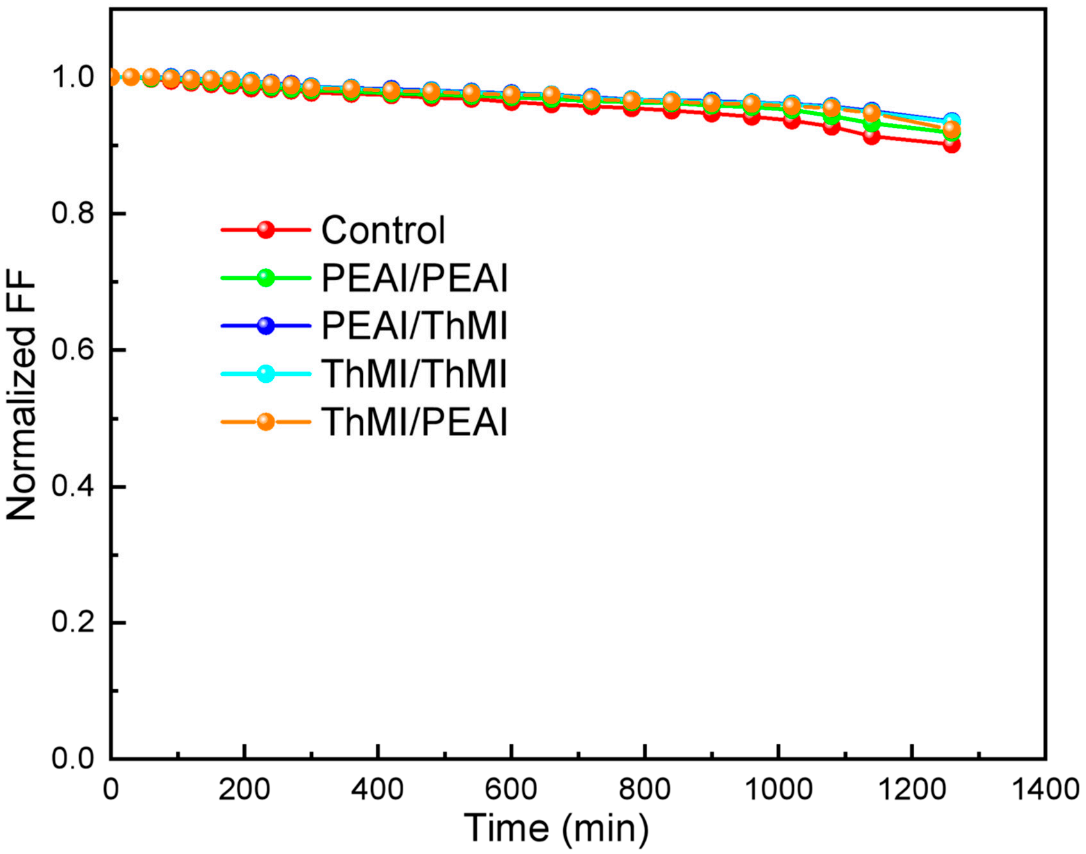

Figure S12. Normalized FF over time for unencapsulated control and dual-interface passivation devices stored under ambient air (RH 30-40%) at room temperature.

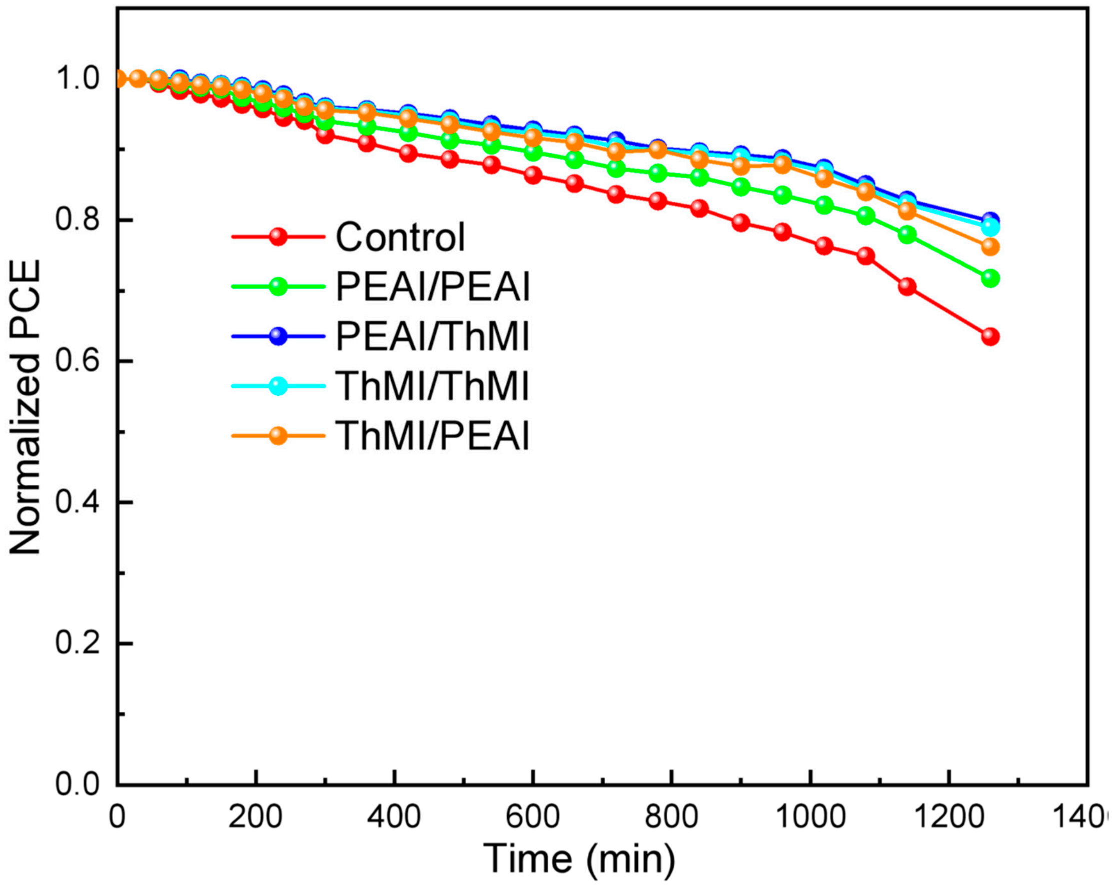

Figure S13. Normalized PCE over time for unencapsulated control and dual-interface passivation devices stored under ambient air (RH 30-40%) at room temperature.
